# Similarities and differences with the ‘general public’: Chinese civil servants’ attitude to genetically modified organisms and its influencing factors

**DOI:** 10.1080/21645698.2023.2256929

**Published:** 2023-09-14

**Authors:** Zheng Yang, Danfeng Liao, Hepeng Jia

**Affiliations:** a The School of Communication, Soochow University, Suzhou, China; b The Deaprtment of Science Popularization, China Association of Agricultural Science Societies, Beijing, China

**Keywords:** Administrative literacy, attitudes to GMOs, belief in conspiracy theories, civil servants, occupational literacy, scientific literacy

## Abstract

This study examines Chinese civil servants’ attitudes toward genetically modified organisms by reviewing a national survey of 3,018 Chinese civil servants. The findings show that Chinese civil servants hold a more positive attitude to GMOs than the wider Chinese “general public”, with a similar level of genetic scientific literacy and belief in GMOs conspiracy theories and their influence mechanisms. While the Chinese civil servants’ occupational literacy plays an important role in their GMOs attitude. This study provides a new mind-set for studying some specific groups’ attitudes toward GMOs and related food policies.

## 1. Introduction

The public’s attitude toward genetically modified organisms (GMOs) has always been a hot topic in the fields of food policy, public management, and food management in both the Western context^1–3^ and the Chinese context.^4,5^ Many studies have already revealed the public’s attitude toward GMOs in different countries (or regions), for instance, Cui and Shoemaker,^6^ found that 11.9%, 41.4%, and 46.7% of the Chinese public have a positive, neutral, or negative view of GM food, respectively (p.1). Ruth et al.^7^ found that most of the American public had positive attitudes toward GM science, according to a national survey (p.113). Hakim et al.^8^ found that due to the inability to effectively identify GMOs labels, most Brazilian consumers (74.6%) hold a negative or conservative attitude toward GMOs. The application and promotion of GMOs have also been found to have a profound impact on politics, social and economics.^9^

Most existing studies about attitudes toward GMOs have focused on the “general public” as a homogenous entity (see)^1–5,10–12^ or unsubdivided consumers.^8,13–20^ However, many scholars have already pointed out that public approaches to GMOs are not homogeneous.^21–23^ Subdivided groups with different demographic characteristics may have different attitudes, views, and behaviors toward GMOs.^24,25^

To date, there has been little systematic research on subdivided groups’ attitudes to GMOs or other potentially controversial food topics and their influencing factors. Although academia seems to have reached a consensus on this view that says the public is no longer homogeneous in their attitudes toward GMOs, the answer to how and why different demographics have different attitudes is still unclear. Among diverse “public groups”, the civil servant group is a special case in relation to GMOs. Because of their occupational attribute as governmental staff, as well as their occupational characteristics of exercising governmental administrative power and performing official duties, civil servants are generally considered to be government “spokespersons” in society.^26^ Their words and deeds are also considered to be characterized by “government credibility”,^27^ which can be used to reduce public fear of and promote GMOs.^13^ Therefore, civil servants’ attitudes toward GMOs, especially those in agriculture and food related departments, have a demonstration and guidance function for the wider society.^28^ There was a saying in China that “civil servants should take the lead in eating genetically modified foods”.^a^^a.^See https://net.blogchina.com/blog/article/1561161 Compared to the general public and other social groups, civil servants and their attitudes are more likely to have an impact on agricultural policy decisions regarding GMOs and other crop products. Therefore, it is necessary to separate civil servants from the “general public” to investigate their understanding and attitude toward GMOs separately, which is the main research objectives of this study. To achieve this objective, a large-scale national questionnaire survey of Chinese civil servants (*n* = 3,018) was conducted to investigate and analyze Chinese civil servants’ attitudes to GMOs and their influencing factors, and also to provide reference for how to study more demographically subdivided publics’ attitudes toward GMOs.

## 2. General Public or Specific Groups? Civil Servants and Their Attitudes to GMOs

Many studies have shown general global consumer attitudes toward GMOs.^1,4,8,10,13–20^ Some have shown that Chinese consumers still hold a relatively negative attitude toward GMOs.^5,6,20^ Such a negative attitude toward GMOs by the Chinese public is considered to be related to Chinese traditional culture (such as advocating natural food); media reports that demonize GMOs; low scientific literacy, especially gene related scientific literacy, among the Chinese public; and China’s long-term top-down science popularization system.^29–32^ As mentioned above, such studies have focused on the “general public” and their attitudes toward GMOs in China. However, as Nisbet and Scheufele^33^ have emphasized, the public are diverse in terms of social backgrounds, which also leads to different reactions and behaviors in the face of food topics such as GMOs. This claim has been supported and expanded upon by many other scholars, for instance, Besley and Nisbet^34^ and Simis et al.,^23^ who both found that treating the public as a homogeneous entity hinders public managers from effectively conveying correct knowledge. Irwin and Michael^35^ put it more directly: “rather than presenting ‘the public’ as a fixed and homogeneous entity, it becomes necessary to view the possibly diverse and dynamic character of public” (p.149). Kyvik^36^ suggested that “the public is not a homogeneous audience” (p.290), and Rose et al.^37^ claimed that, “a homogeneous public does not exist” (p.261).

However, in studies on public attitudes toward GMOs, researchers do not seem to have fully applied these conclusions. Many studies still regard non-scientist citizens as a homogeneous “public” group and do not further subdivide them, whether from an occupational perspective or other demographic perspectives (see).^1–5,12,38,39^ In China, only a very small number of studies on public attitudes toward GMOs have focused on farmers;^40–42^ nearly all other studies are aimed at the unsubdivided “general public” and use the terms “general public” or “consumer.”^5,6,43–46^ This may be related to the specific situation of science communication in China, known as “science popularization.” Due to its political and utilitarian characteristics, the object of “science popularization” in China is a unified group that receives education from scientists to improve their scientific literacy,^31,47^ which is more like the “one-way discourse aimed at an unrealistically homogeneous public.”^48^

The reality is that the subdivided groups, with their different demographic characteristics, may have very different attitudes, views and behaviors around GMOs.^24,25^ For instance, several studies have already pointed out that, compared with male consumers, female consumers generally have a more negative attitude toward GMOs;^49,50^ those with different religious beliefs and income levels also have different attitudes toward GMOs.^51^ However, these studies have not focused on a specific group, particularly in relation to occupation. Different occupations will lead to different exposure to and understanding of GMOs. For example, farmers are often exposed to genetically modified crops, so their attitudes toward GMOs are more nuanced or extreme than those of the general public.^40,42^

Among all occupations, civil servants are special. Because of their occupational identity as governmental staff, as well as their characteristics of exercising governmental administrative power and performing official governmental duties, civil servants are generally considered to be the “spokespersons” of governments in society.^26^ Their words and deeds are also considered to characteristics that are imbued with “government credibility”.^27^ Such professional attributes make civil servants’ attitudes affect the attitudes of other public groups in society.^52^ For instance, civil servants’ understanding and practice of morality has been found to play an effective demonstration and leadership role for the public.^53^ When faced with potentially controversial scientific and technological issues, civil servants’ attitudes and behaviors also hold a demonstration function for the public. For example, Gamson and Modigliani^54^ found that when the media played up the general trust of government personnel in nuclear power, the public’s attitude toward nuclear power also improved.

It can thus be speculated that the attitude of civil servants will have an impact on the attitude of other citizens on the issue of GMOs. In China, there are already certain voices among the citizens who say that “civil servants should take the lead in eating genetically modified foods” as mentioned above. In addition, the occupational attributes of civil servants, especially in China, requires them to be loyal and obedient to national policies, as in the *Decision on Amending the Administrative Measures for the Safety Evaluation of Agricultural Genetically Modified Organisms (Exposure Draft)* released by the Ministry of Agriculture and Rural Affairs of the People’s Republic of China, which aimed at promoting the industrialization of genetically modified crops. Such occupational characteristics and national policies will also affect civil servants’ attitudes to GMOs, making them different from the general public’s. Therefore, it is necessary to separate civil servants from the “general public” to investigate their GMOs cognition and attitude separately. The following research questions are thus proposed:

RQ1: What is the attitude of Chinese civil servants toward GMOs?

RQ2: What is the difference between Chinese civil servants’ attitudes to GMOs and those of the general Chinese public?

## 3. Scientific Literacy, Occupational Literacy, Belief in Conspiracy Theories, and Attitude to GMOs

Scientific literacy is a factor affecting the public’s attitude toward GMOs. Although the knowledge-attitude model in public understanding of science has been widely criticized^55,56^ and many studies have already shown that there is no simple positive linear relationship between the public’s scientific knowledge (scientific literacy) and their attitude toward science^55,57–60^ when it comes to specific GMOs issues, many studies have found that the public’s scientific knowledge (especially gene-related) can significantly positively affect their attitude toward GMOs.^8,14,17,30,32^ However, some studies have found that more knowledge, including specific biotechnology knowledge about GMOs, does not result in a higher level of acceptance of GM foods^5,61^ and could even led to more polarized beliefs.^62^ Facing other controversial scientific issues, some studies have found that although general scientific literacy does not play a significant role on scientific attitude, the specific scientific literacy related to those issues can play a positive role on their attitude toward them, such as vaccine scientific literacy and vaccination attitude.^63^ It appears that the impact of public scientific knowledge/literacy on people’s attitudes toward GMOs is complex and elusive.^64^ Such a complex relationship may also be related to the diversity of the public. As discussed above, the public is not a unified group; different groups may have completely different attitudes and understandings of a specific scientific topic which may further influence the mechanism of scientific literacy on GMOs. Almost all the above studies take the so-called “general public” as their research object without further subdivision.^5,17,30,32,61^ It is important to subdivide the public to further study the impact of scientific literacy on attitudes to GMOs, to better understand the relationship between knowledge and attitude in relation to some specific scientific issues. Therefore, the following research question is proposed:

RQ3: What is the relationship between the GMOs scientific literacy of Chinese civil servants and their attitude to GMOs?

When the public is subdivided as the research object, the special group attributes of these subdivided groups are particularly important. For instance, when studying the attitude of millennials to GMOs, their historical context and value characteristics are important factors.^65^ When using occupation as the subdivision standard, those occupation-related factors, such as occupational literacy, become of great analytical value, as does scientific literacy. Occupational literacy relates to the skills and abilities employees need to have to be successful at work and to manage the demands of their jobs in a healthy, productive way.^66^ For civil servants, their occupational literacy is embodied in “administrative literacy”.^67^ Civil servants’ administrative literacy is different from citizens’ administrative literacy, which highlights citizens’ capacity to obtain, process, and understand basic information and services from public organizations needed to make appropriate decisions,^68^ but emphasizes the understanding and executive ability of civil servants for administrative work.^67^ Occupational literacy is considered to have a profound impact on the daily life and work of the occupational group, and even on their psychological state and self-cognition in both working and non-working situations^69^ – civil servants’ occupational literacy has been found to have a significant impact on their self-identity,^67^ which may further affect their views on GMOs. To date there has been no systematic study exploring the role of occupational literacy in terms of attitude to GMOs of specific occupational groups. Therefore, the following research question is proposed:

RQ4: What is the relationship between the occupational (administrative) literacy of Chinese civil servants and their attitude to GMOs?

In addition to the literacy dimension, many studies have found that public belief in conspiracy theories will significantly affect their attitude toward specific scientific and technological issues, such as vaccinations,^63,70,71^ climate change,^72–74^ and GMOs,^75–77^ in both Western and Chinese contexts. According to Yang,^78^ there are four kinds of main GMOs conspiracy theories in China: GMOs as biological weapons; GMOs as state control tool; business conspiracy theories; and genocide conspiracy theories. The more inclined the public is to believe these GMOs conspiracy theories, the more likely they are to have a negative attitude toward GMOs.^6,75,76^ Therefore, reducing the public’s belief in GMOs conspiracy theories has become an effective means to improve the public’s attitude toward GMOs.^6^ Unfortunately, with the rapid development of the digital media environment, conspiracy theories have not yet been effectively curbed, even with a growing trend in many scientific affairs including GMOs.^79^ In addition, many studies have found that belief in GMOs conspiracy theories is widespread in all social classes and occupations,^74,79^ especially in the Chinese context.^6,78^ Therefore, we have reason to speculate that even civil servants would believe GMOs conspiracy theories, which may further affect their attitude toward GMOs. Thus, the following research questions are proposed:

RQ5: For Chinese civil servants, what impact does their belief in GMOs conspiracy theories and scientific literacy have on their attitude to GMOs?

## 4. Method

### 4.1. Sampling and Data

When investigating attitudes toward GMOs, surveys are widely used instruments (see)^1,4,8,10,13–20^ which can effectively help understand the attitudes and perceptions of a large number of research subjects toward a specific issue. Thus, a national survey of Chinese civil servants was conducted with the help of the China Association of Agricultural Science Societies and provincial Agricultural and Rural Departments and local Association of Agricultural Science Societies in China during March and June 2022. The average length of time to complete the questionnaire was 25 minutes. The survey was distributed to seven provinces in China, covering both agricultural and non-agricultural provinces, and encompassed the three most important regional classification standards in China: the eastern, central, and western regions. A total of 3,200 questionnaires was distributed, and 3,018 valid questionnaires were received, with a recovery efficiency of 94.3%. Although this number is small compared to the 7.1 million civil servants in China, it represents similar demographics in terms of gender, age, and administrative level to the overall civil servant group in China, and therefore has a certain degree of representativeness. Among the 3,018 civil servant participants, 59.5% (*N* = 1,796) were male and 40.5% (*N* = 1,222) female. Most were born in the 1970s (36.1%) and 1980s (25.8%). More demographic information is shown in Table 1.

**Table 1.** Demographic information of participants.

		Proportion/%			Proportion/%
Gender	Male	59.5	Education background	Liberal arts	14.5
	Female	40.5		Science and technology	19.4
Age	1950s	2.0		Medicine	1.9
	1960s	18.7		Agronomy	51.3
	1970s	36.1		Others	13.0
	1980s	25.8	Administrative level	Deputy-Bureau-Director level and above	0.1
	1990s	17.4		Division-Head level	1.0
Education level	Master’s and above	9.0		Deputy-Division-Head level	2.9
	Bachelor’s	61.4		Section-Head level	12.3
	Junior college	23.6		Deputy-Section-Head level	15.3
	Senior high school	5.5		Staff level	34.2
	Junior high school and below	0.4		Clerk level	34.2
Area	Eastern Region	35.7		Others	0
	Midland	40.1			
	Western Region	24.2			

### 4.2. Operationalization of Key Variable

The questionnaire used in this study focused on seven sections, covering a total of 28 questions and 87 items: (1) administrative literacy of Chinese civil servants; (2) the attitude of Chinese civil servants toward GMOs; (3) Chinese civil servants’ GMOs scientific literacy; (4) the participation of Chinese civil servants in GMOs science popularization activities; (5) the attitude of Chinese civil servants toward GMOs industrialization; (6) the nationalism of Chinese civil servants; (7) Chinese civil servants’ attitudes toward public participation in GMOs issues. This study mainly used the data from the first three parts. Items were adapted from Cui and Sharon,^43^ and You and Jin^80^ to measure Chinese civil servants’ attitudes toward GMOs on an 11-point scale where 0 means extremely negative attitude and 10 means extremely positive attitude. The civil servants’ beliefs were tested by five GMOs conspiracy theories that are widespread in China, with results from 1 (mostly disagree) to 5 (mostly agree); the higher the score, the more they believe in the conspiracy theories. Items used by Tianjin Personnel Bureau & Tianjin Institute of Administration National Civil Service Training Needs Research Group,^81^ and Duan and Liu^67^ on the administrative ability literacy of civil servants in China were used to measure the civil servants’ administrative literacy and contained 25 items. The results of Chinese civil servants’ administrative literacy are presented as a 5-point scale, where the higher the score, the higher the literacy. This study did not adopt traditional scientific literacy survey questions, but eight questions related to genetic science were used to measure Chinese civil servants’ GMOs-related scientific (genetic) literacy,^82^ since specific scientific literacy has been found to be more related to peoples’ attitude toward specific scientific issues^63^ the results are presented as an 8-point scale, where the higher the score, the higher the literacy. The study did not use the general scientific literacy test because many scholars have already pointed out that the specific scientific literacy related to a special scientific issue often plays a more obvious role than general scientific literacy on that issue.^64,83^ The descriptive data for these variables is summarized in Table 2.

**Table 2.** Descriptive statistics of key variables.

Variables	*M*	*SD*	*α*
Attitude to GMOs	7.56	2.44	.77
Belief in GMOs conspiracy theories	2.73	1.29	.75
GMOs-related scientific literacy (genetic literacy)	4.81	2.22	.80
Administrative literacy	4.37	0.41	.69

## 5. Analysis and Results

### 5.1. Similar to General Public Group: Chinese Civil servants’ Attitudes, Conspiracy Theory Beliefs, and Scientific Literacy of GMOs

Until 2020, the planting area of GM crops in China encompassed only 3.2 million hectares, far lower than countries such as the United States (71.5 million hectares) and Brazil (52.8 million hectares). Although the Chinese government issued a series of policies to promote the development and industrialization of GMOs, such as the *Decision on Amending the Administrative Measures for the Safety Evaluation of Agricultural Genetically Modified Organisms (Exposure Draft)* released by the Ministry of Agriculture and Rural Affairs of the People’s Republic of China, and standardized the marketization of GMOs, such as *Regulations on the Safety Management of Agricultural Genetically Modified Organisms* released by The State Council of China, the general public in China continues to show a lack of trust and low consumption tendency toward genetically modified crops and food. According to Cui and Shoemaker,^6^ only 11.9% of the Chinese public has a positive view of GMOs. Wu and colleagues^93^ found that only 11.5% of the Chinese public choose to actively purchase genetically modified foods. Overall, the Chinese public’s attitude and willingness to consume GMOs are not optimistic. But the survey results in this study show that Chinese civil servants have a relatively positive attitude toward GMOs (*M = 7.56, SD = 2.44*), which is significantly more positive than the “general public” in China according to the above surveys and their review of Chinese public attitudes toward GMOs^6^ Wu et al.,)^93^.

But this kind of positive attitude is not reflected in all aspects of GMOs. The survey results also show that only 16.3% of civil servant respondents stated that they would actively purchase GMOs (*M = 2.28, SD = 1.308*). But in supporting the country’s active research of GMOs, as well as government management, they have shown a very clear supportive attitude (*M = 3.66, SD = 1.412; M = 3.80, SD = 1.384*) (Table 3). On the issue of GMOs there is a clear binary differentiation between individual behavioral attitudes and national behavioral attitudes among Chinese civil servants. At the level of national research and control, the civil servant group shows a supportive attitude that is relatively consistent with national policy guidelines, influenced by their professional attributes, but at the level of personal consumption behavior, the civil servant group still holds a relatively conservative attitude.

**Table 3.** Chinese civil servants’ attitudes toward GMOs-related issues.

	Least Agreed（1）	Not Agreed（2）	Neutral（3）	Agreed（4）	Most Agreed（5）	M	SD
I will proactively purchase GMOs	42.5%	11.9%	29.3%	8.0%	8.3%	2.28	1.308
I support scientific research in GMOs	13.2%	6.7%	22.7%	15.3%	42.1%	3.66	1.412
I support GMOs government management	11.8%	5.5%	20.6%	15.3%	46.9%	3.80	1.384

The survey data also shows that the average score of the Chinese civil servants’ conspiracy theories belief (*M = 2.75, SD = 1.29*) exceeds the median score (2.5), that is, Chinese civil servants are more inclined to hold a neutral or positive attitude toward GMOs conspiracy theories than to dismiss them. This is similar to the survey results of Cui and Shoemaker^43^ on the Chinese general public’s beliefs in GMOs rumors and conspiracy theories. According to Cui and Shoemaker,^43^ the Chinese public tends to believe or has a neutral attitude toward nonscientific politicized or commercialized GMOs conspiracy theories, such as “GMOs is a huge conspiracy and a biological weapon against developing countries”. The results also show that the average genetic science literacy score of the Chinese civil servant sample is 4.81 (*SD = 2.219*). Samples with scores of 4 and below accounted for 41.1% of the total, of which 5.3% did not answer one question correctly, while the samples with all pairs accounted for 11.1% of the total. The overall genetic science literacy of civil servants is therefore limited, with no clear difference from the general public in level of transgenic knowledge, although Chinese civil servants’ genetic science literacy is a little higher than the general publics’, according to Chen and colleagues’ results.^84^ Furthermore, based on a series of hierarchical regression models, Chinese civil servants’ belief in GMOs conspiracy theories significantly negatively affects their attitude toward GMOs, while their genetic scientific literacy significantly positively affects their attitude (Table 4), which provides answers for RQ3 and RQ4. This is also consistent with many studies on factors influencing the general public’s attitude toward GMOs, especially focusing on the Chinese publics.^17,30,32,43,75,76,84^

**Table 4.** Hierarchical regression predicting Chinese civil servants’ attitude toward GMOs.

Variables	Model 1	Model 2	Model 3	Model 4
Block 1: Control variables				
Gender	−.87	−1.62	−1.26	−.53
Age	1.33	.25	.46	−1.56
Education level	.21	1.20	2.32*	2.70**
Education background	2.44*	2.55*	2.30*	2.23*
Administrative level	−.74	−.41	−.15	−.31
Total *R*^*2*^	9.1%	9.1%	9.1%	9.1%
Block 2: Conspiracy theory beliefs				
Conspiracy theory beliefs		−10.61***	−7.25***	−7.43***
Total *R*^*2*^		21.0%	21.0%	21.0%
Block 3				
Genetic scientific literacy			8.31***	8.83***
Total *R*^*2*^			25.6%	25.6%
Block 4				
Administrative literacy				12.62***
Total *R*^*2*^				33.5%

Figures in the table are *N*=3,018. Cell entries are standardized regression coefficients. Gender (1 = male, 0 = female); Education background (0=Other; 1=Liberal arts; 2=Science and technology; 3= Medicine; 4= Agronomy). *p < .05. **p < .01. ***p < .001.

For RQ1 and RQ2, the above analysis shows that Chinese civil servants have similar levels of belief in GMOs conspiracy theories and genetic scientific literacy as the Chinese “general public”, as well the affecting mechanism; but Chinese civil servants show a significantly more positive attitude than the general public.^6^ This indicates that civil servants are a special group when it comes to GMOs – there must be factors driving a more positive attitude toward GMOs among civil servants. Moreover, such difference in attitude is not caused by their scientific literacy of GMOs and conspiracy theory beliefs, which are like those of the public; there must be factors that specifically belong to this group that affect their attitude toward GMOs. In summary, facing the GMOs issue, there are similarities between Chinese civil servants and the general public, such as their levels of belief in GMOs conspiracy theories and genetic scientific literacy, but there are also significant differences between the two groups: the civil service community generally has a more positive attitude toward GMOs than the Chinese general public. Finding the reasons for this “different and more positive attitude” is the key to the subsequent analysis of this article.

### 5.2. A Special Public Group: Chinese Civil servants’ Administrative Literacy and Its Mediation Effects

As discussed above, Chinese civil servants represent a special group that is similar but different from the general public in relation to the issue of GMOs, and certain factors that specifically belong to this group may affect their more positive attitude toward GMOs. This characteristic may be related to their special professional attributes and the occupational literacy required by the profession. Therefore, the relationship between their occupational (administrative) literacy and their attitude toward GMOs, conspiracy theories belief, and scientific literacy is further analyzed. Firstly, as per Table 3, it is clear that Chinese civil servants’ administrative literacy is a significant positive predictor of their attitudes toward GMOs (*β* = 12.62, *p* < .001), which means that those Chinese civil servants with higher occupational literacy tend to have a more positive attitude toward GMOs. This may be due to the strong requirements of logic and the ability to collect information and discriminate between it in the administrative literacy of Chinese civil servants, so the higher the administrative literacy civil servants have, the more effective they can obtain and judge the correct scientific information about GMOs, thus generating a more positive attitude toward GMOs.

**Figure 1.**
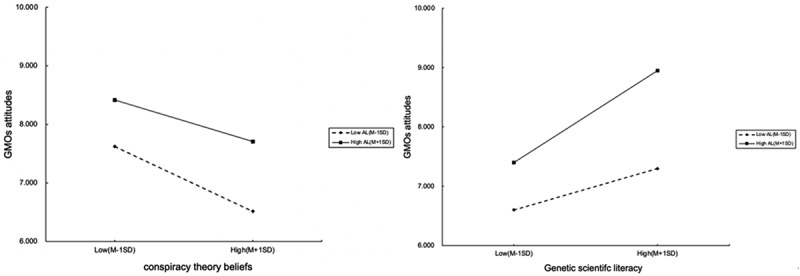
Mediation of administrative literacy on Chinese civil servants’ attitude to GMOs.

Furthermore, through the Simple Slope Test of Regression Analysis, it can be found that as a mediation factor, the occupational (administrative) literacy of Chinese civil servants significantly adjusted the impacts of their GMOs conspiracy theories beliefs and genetic scientific literacy on their attitude toward GMOs (Fig. 1). For those Chinese civil servants with higher administrative literacy, their attitude to GMOs is less negatively affected by their belief in GMOs conspiracy theories, and the positive impact of their genetic scientific literacy on their attitude to GMOs can be effectively amplified compared to those with lower administrative literacy. Combined with the findings in Table 3, the answer to RQ5 is that the occupational literacy of Chinese civil servants plays a very important role in their attitude toward GMOs. It can not only directly positively affect Chinese civil servants’ attitudes to GMOs, but through moderation effects it can also amplify the positive effect of genetic scientific literacy and reduce the negative effect of conspiracy theory beliefs on their attitudes to GMOs. These findings further provide a deeper understanding for RQ3 and RQ4 that as a special public group the civil servants’ particularity – their occupational characteristics and literacy – can play an important role in their attitudes to GMOs. This may also be one of the reasons why the Chinese civil servants’ attitudes to GMOs are different to those of the general public in China.

## 6. Discussion: Investigating Attitudes to GMOs with a Specific Public Group

This study has revealed Chinese civil servants attitudes to GMOs and their influencing factors: their belief in GMOs conspiracy theories and genetic scientific literacy, which is similar to the general public and which significantly negatively and positively influences their attitude to GMOs respectively; and their occupational literacy, which not only directly positively affects Chinese civil servants’ attitude to GMOs, but also through moderation effects, amplifies the positive effect of genetic scientific literacy and reduces the negative effect of conspiracy theory beliefs on their attitudes to GMOs. The reason the administrative literacy of civil servants has such significant impact on their attitude to GMOs has two possible answers. Firstly, the administrative literacy level was found to be significantly proportional to the civil servants’ support for national policies.^85^ China’s national policies are biased toward supporting GMOs – the government issued policies and regulations to promote the industrialization of GMOs in 2022.^86^ Therefore, it is understandable that for Chinese civil servants, the higher their administrative literacy, the more positive is their attitude to GMOs. Secondly, administrative literacy is a multi-dimensional indicator. The measures used in this study include some science related self-evaluation, such as “I think civil servants should have complete theoretical knowledge, such as general science” and “I think civil servants should have a wealth of knowledge”, as well as an ability to engage in self-evaluation, such as “I have clear criteria for judging whether it is good or evil” and “I judge things by scientific standards rather than human feelings”. In the administrative literacy test, there are many items related to the ability of logic and information collection and discrimination. Therefore, it can be speculated that civil servants with high administrative literacy may also have high scientific judgment ability, which can not only positively influence their attitude to GMOs, but also moderates the effect of conspiracy theories beliefs and genetic scientific literacy on their attitude to GMOs.

The main contribution of this study is to respond to many scholars’ conclusions that the public is homogeneous in terms of food policy and management with an empirical and realistic case study to prove them wrong.^21–23^ As a special group, civil servants share similarities and differences with the wider public group in terms of attitude to GMOs, conspiracy theory beliefs and genetic scientific literacy and they have a more positive attitude toward GMOs than the general Chinese public. Highlighting the special occupational attributes and literacy of Chinese civil servants shows that the special attributes (such as occupation) of those special groups may play an important role in the formation of their attitudes to GMOs and other scientific issues. As Irwin and Michael^35^ observed, “rather than presenting ‘the public’ as a fixed and homogeneous entity, it becomes necessary to view the possibly diverse and dynamic character of public” (p.149). We need to pay more attention to the group/professional attributes of heterogeneous public groups and the role of such specific attributes in food management, food attitudes and more, which could start by avoiding the term “general public” in research on GMOs attitudes, especially in China, a country with a vast and diverse population. The formation of the public’s attitude to GMOs is affected by many diverse personal and societal factors.^87,88^ When research objects (the general public) are subdivided, the role of personal and social factors in the formation of attitudes to GMOs can be understood more clearly and may lead to new ideas and unexpected conclusions. As Jasanoff^89^ states, “publics are not all alike but are guided by culturally conditioned ‘civic epistemologies’” (p.23). The administrative literacy of civil servants provides a case study for such “civic epistemologies” of different public groups.

The findings around the influence of occupational literacy on civil servants’ attitudes to GMOs further indicates that there may be ways to improve attitudes to GMOs from the perspective of professional quality. Many scholars have shown that conspiracy theories about GMOs are becoming more and more popular, especially in the digital media environment.^75–77^ It is hard to reduce the public’s belief in GMOs conspiracy theories in the current media environment.^90,91^ The finding that civil servants’ occupational literacy can significantly reduce the negative effect of conspiracy theory beliefs on their attitude to GMOs provides a new perspective: if we cannot effectively reduce the GMOs conspiracy theory beliefs of a specific occupational group directly, improving their occupational literacy to reduce the negative impact of their beliefs on their attitudes to GMOs may be another feasible path. Cultivating occupational literacy seems to be an easier route for a specific occupational group in a working environment.^92^ This can also provide a new perspective for studying the conspiracy theory beliefs of other special groups.

This study also has referential significance for China’s agricultural departments. In the current process of promoting the industrialization of GMOs in China, the civil service community can impact the wider acceptance of GMOs among Chinese public. Increasing the acceptance of GMOs among Chinese civil servants may positively affect the acceptance of GMOs among the Chinese public. Thus, in addition to traditional methods, such as improving civil servants’ scientific literacy and reducing their conspiracy theory beliefs, based on this study, improving the professional occupational literacy of civil servants is an innovative measure. This also provides some future research directions, such as how to effectively enhance the civil servants’ GMOs attitudes through improving their occupational literacy, and how to use the GMOs attitudes of civil servants to influence the broader public’s attitudes toward GMOs.

## 7. Conclusion

This study investigated Chinese civil servants’ attitudes to GMOs and their influencing factors, mainly focusing on their occupational (administrative) literacy. The findings indicate that as a special group, Chinese civil servants hold a more positive attitude toward GMOs than the wider public’s, with a similar level of genetic scientific literacy and GMOs conspiracy theories belief influencing their attitudes to GMOs. The particularity of this group is reflected in the impact of their special occupational literacy on their attitudes to GMOs, which can not only directly positively affect Chinese civil servants’ attitudes to GMOs, but also through moderation effects, amplify the positive effect of genetic scientific literacy and reduce the negative effect of conspiracy theory beliefs. This study provides an empirical case to suggest that “the public is not a fixed and homogeneous entity but many diverse and dynamic groups”,^1–5,12,35,38,39^ as well as suggesting a new mind-set for improving some specific groups’ attitudes to GMOs.

There are also some limitations in this study. For instance, the civil servant group is particular among the non-scientist public, such as their relationship with the government, especially in China, an authoritarian political system. Therefore, the applicability of this study to other types of subdivided public may also be relatively limited and needs to be supplemented by further empirical research.
